# Understanding the Variability of 22q11.2 Deletion Syndrome: The Role of Epigenetic Factors

**DOI:** 10.3390/genes15030321

**Published:** 2024-02-29

**Authors:** Francesca Cillo, Emma Coppola, Federico Habetswallner, Francesco Cecere, Laura Pignata, Elisabetta Toriello, Antonio De Rosa, Laura Grilli, Antonio Ammendola, Paolo Salerno, Roberta Romano, Emilia Cirillo, Giuseppe Merla, Andrea Riccio, Claudio Pignata, Giuliana Giardino

**Affiliations:** 1 Department of Translational Medical Sciences, Pediatric Section, University of Naples Federico II, 80138 Naples, Italy; francescacillo97@gmail.com (F.C.); emmacopp24@gmail.com (E.C.); federico.habetswallner@gmail.com (F.H.); betty.toriello@gmail.com (E.T.); antonioderosa06@libero.it (A.D.R.); lauragrilli98@gmail.com (L.G.); roberta.romanomd@gmail.com (R.R.); emiliacirillo83@gmail.com (E.C.); giuliana.giardino@unina.it (G.G.); 2 Department of Environmental Biological and Pharmaceutical Sciences and Technologies, Università degli Studi della Campania “Luigi Vanvitelli”, 81100 Caserta, Italy; francesco.cecere@unicampania.it (F.C.); laura.pignata@unicampania.it (L.P.); andrea.riccio@unicampania.it (A.R.); 3 Department of Molecular Medicine and Medical Biotechnology, University of Naples Federico II, 80138 Naples, Italy; antonio.ammendola2@studenti.unina.it (A.A.); paolo.salerno@unina.it (P.S.); giuseppe.merla@unina.it (G.M.); 4 Laboratory of Regulatory and Functional Genomics, Fondazione IRCCS Casa Sollievo della Sofferenza, 71013 San Giovanni Rotondo, Italy

**Keywords:** 22q11.2 deletion syndrome, epigenetics, micro-RNAs, methylation, CpG islands

## Abstract

Initially described as a triad of immunodeficiency, congenital heart defects and hypoparathyroidism, 22q11.2 deletion syndrome (22q11.2DS) now encompasses a great amount of abnormalities involving different systems. Approximately 85% of patients share a 3 Mb 22q11.2 region of hemizygous deletion in which 46 protein-coding genes are included. However, the hemizygosity of the genes of this region cannot fully explain the clinical phenotype and the phenotypic variability observed among patients. Additional mutations in genes located outside the deleted region, leading to “dual diagnosis”, have been described in 1% of patients. In some cases, the hemizygosity of the 22q11.2 region unmasks autosomal recessive conditions due to additional mutations on the non-deleted allele. Some of the deleted genes play a crucial role in gene expression regulation pathways, involving the whole genome. Typical miRNA expression patterns have been identified in 22q11.2DS, due to an alteration in miRNA biogenesis, affecting the expression of several target genes. Also, a methylation epi-signature in CpG islands differentiating patients from controls has been defined. Herein, we summarize the evidence on the genetic and epigenetic mechanisms implicated in the pathogenesis of the clinical manifestations of 22q11.2 DS. The review of the literature confirms the hypothesis that the 22q11.2DS phenotype results from a network of interactions between deleted protein-coding genes and altered epigenetic regulation.

## 1. Introduction

Briefly, 22q11.2 deletion syndrome (22q11.2DS) is a complex and heterogeneous clinical syndrome. Under the 22q11.2DS definition are included several phenotypes such as the historically known DiGeorge syndrome (DGS), velocardiofacial syndrome (VCFS) and conotruncal anomaly face syndrome (CTAF) [1]. The primarily acknowledged presentation is the classic clinical triad including congenital heart defects (*CHD*) (75% of patients), T-cell compartment immunodeficiency due to hypoplastic/aplastic thymus (75% of patients) and hypocalcemia due to the developmental defect of parathyroid glands in 50% of cases, formerly referred to as DGS [1]. This term is now used for those individuals who show clinical phenotype of 22q11.2DS in the absence of an identified 22q11.2 deletion, in whom alternative pathogenetic alterations occur. Briefly, 22q11.2DS represents the most frequent microdeletion syndrome observed in the human genome, with an estimated incidence of 1 in 1.000 fetuses [2,3] or approximately 1 in 3.000–6.000 newborns [1]. However, a recent study conducted using DNA samples from dried blood spots for newborn screening reports an estimated minimum 22q11.2 DS prevalence of 1 in 2.148 live births [4]. Despite the significant incidence, no routine approach to prenatal screening for this condition has been established [5,6,7,8]. Newborn screening to measure the number of TREC copies successfully identifies 22q11.2 DS with T-cell lymphopenia, which can be helpful to prevent subsequent complications such as hypocalcemia [9,10]. In 90% of cases, the 22q11.2 deletion occurs de novo during gametogenesis as a consequence of nonallelic homologous recombination events. In 10% of patients, the syndrome is inherited in an autosomal-dominant fashion. The predominance of de novo cases may be partially explained by the impaired reproductive fitness of the patients carrying the deletion [11], especially males [12]. This hypothesis is also supported by the evidence that, in the familial forms, the disease is usually inherited from the mother [13,14]. Apart from the most recognizable aspects, more than 180 different phenotypic features have been described [15,16,17] in 22q11.2DS patients, and the syndrome is characterized by the extreme variability of the type and severity of the clinical manifestations, which can be also observed in members of the same family [1,15,18,19,20,21,22]. The phenotypic variability consists of a different combination of clinical manifestations, which compose a syndromic picture with various degrees of severity. Moreover, the same clinical abnormality can vary from mild to life-threatening in different subjects [23,24,25,26]. The syndrome can be associated with different size 22q11.2 region deletions. However, there is no correlation between the extension of the deletion and the severity of the syndrome [27]. Interestingly, microduplications of the 22q11.2 region result in a syndrome characterized by developmental delay, congenital heart defects, craniofacial dysmorphisms, behavioral alterations, visual and hearing impairment, and urogenital abnormalities, presenting with great clinical variability and absent genotype–phenotype predictability [28,29,30]. In this review, we summarize the main genetic and epigenetic mechanisms that may underlie the clinical variability in 22q11.2DS.

## 2. Main Clinical Features

Congenital heart defects (CHDs) are observed in up to 75% of the patients and represent the main cause of mortality in young patients affected by 22q11.2DS (87%). The most frequent abnormalities are conotruncal heart defects involving the outflow tract such as tetralogy of Fallot, truncus arteriosus, interrupted aortic arch type B (between the left carotid and subclavian arteries) and ventricular septal defect [31]. Pulmonary artery hypoplasia and discontinuity, and aortic arch defects can be present as isolated manifestations (40%) or in association with conotruncal abnormalities (60%) [32]. CHDs are usually diagnosed in the prenatal approach or as one of the first manifestations during the neonatal period [33]. Conotruncal heart defects usually undergo early surgical repair, which requires complex perioperative management in order to lower the complication risks that may derive from comorbidities of the syndrome (e.g., hypocalcemia or airway anomalies) and from the inherently complex cardiovascular anatomy [31,34]. Long-term surveillance is required for all 22q11.2DS patients, not only for those who undergo surgical intervention. In fact, arrhythmias and aortic root dilation have been identified in the absence of CHDs, and long-life risk factors for CHDs including hypertension, obesity and hyperlipemia may also arise during follow-up [35,36].

Palatal abnormalities reported in individuals affected by 22q11.2 DS include a range of defects with variable severity. Only 11% of the children present overt cleft palate, while milder defects like submucosal cleft palate (SMCP), bifid uvula and velopharyngeal dysfunction [37] affect almost 65% of patients. These abnormalities may lead to prenatal nasal regurgitation, and later may lead to nasal emissions or even to the impairment of articulation and the worsening of speech intelligibility in children [38,39].

Due to the absence of the parathyroid glands, up to 50–65% of patients may experience hypocalcemia manifesting with paresthesia, muscle spasms, cramps, tetany, circumoral numbness and seizures [40]. These symptoms represent red flags for the diagnosis of 22q11.2DS. Given their impact, such endocrinological aspects should be routinely monitored, assessing relevant biochemical parameters including parathyroid hormone, calcium or ionized calcium, magnesium and 25-hydroxy vitamin D [41,42] at least annually. In the pediatric population, if calcium levels are low or the dietary intake is not sufficient, supplementation therapy based on calcium and vitamin D should be taken into account whereas daily vitamin D is recommended for all adult patients, reserving calcitriol for refractory cases in both age categories. Overcorrection should be avoided as to prevent hypercalcemia, nephrolithiasis and, ultimately, renal failure. Hence, kidney function, urinary calcium and renal ultrasound should be regularly monitored as well [43]. Other endocrinological manifestations include hypothyroidism in children and in 20% of adults (later-onset manifestation), and hyperthyroidism in children and in 5% of adults; thus, thyroid-stimulating hormone and free thyroxine should be assessed at least annually and, generally, standard treatment is effective [44]. Short stature has been described in 15% of patients, while GH-deficiency and intra-uterine growth retardation have been reported in 4% [45,46].

Immunological features of 22q11.2DS are very heterogeneous, ranging from a completely normal T-cell compartment to severe combined immunodeficiency (SCID) [47]. Most of the patients have moderate T-cell deficiency and are defined as having partial DGS (pDGS). In some cases, a variable association of reduced serum IgG, IgA and IgM levels with reduced vaccines response such as anti-tetanus, anti-diphteria and anti-pneumococcus can be observed [48]. Less than 0.5–1.5% of patients suffer from complete DGS (cDGS) characterized by severe T-cell deficiency, reduced TRECs (T-cell receptor excision circles) and an absent response to mitogens recapitulating the clinical and laboratory features of SCID [49,50]. Recently, data on treatment with cultured thymus tissue (formerly known as thymus transplant) in patients with cDGS are being gathered; in a trial on 105 subjects, a 1-year survival rate of 77% was achieved, along with a T-cell production, developing 6 to 12 months after the procedure, sufficient to provide adequate immune function to prevent serious infections [47,51]. Atypical cDGS has been observed in some cases, and it is characterized by rash, lymphadenopathy and enteropathy arising from the oligoclonal expansion of memory T-cells CD45RO+ [52]. The patients may suffer from recurrent respiratory tract infections. However, previous studies suggest that the susceptibility to recurrent infections is primarily related to the anatomical alterations associated with the syndrome [53] {Giardino, 2019 #1}. Patients with 22q11.2DS may also develop immune dysregulatory manifestations [54] including allergy and asthma, autoimmune diseases such as juvenile idiopathic arthritis [55], hemolytic anemia [56], idiopathic thrombocytopenia [57], autoimmune thyroid dysfunction [58,59] and others.

Developmental delay with gross and fine motor difficulties, articulation and speech abnormalities are also reported in pediatric patients, as well as increased risk for psychiatric disorders such as anxiety, attention deficit, autism spectrum disorders, usually appearing during adulthood. Since 25% of patients are diagnosed with schizophrenia, 22q11.2DS is considered the main genetic predisposition for the development of this disorder [60,61]. From the neurological point of view, 22q11.2DS is also associated with increased risk of provoked and unprovoked seizures and movement disorders like dystonia, myoclonus, and parkinsonism. In particular, provoked (hypocalcemic) neonatal seizures and hypotonia are very early signs [62].

Skeletal alterations including kyphoscoliosis, syndactyly or polydactyly, foot abnormalities and arthropathies are important findings in 22q11.2 DS patients. The reported prevalence of at least one cervical or occipital anomaly is 90.5–100% [63].

Around 60% of patients experience mild-moderate gastrointestinal symptoms such as abdominal pain, gastro-esophageal reflux disease (GERD), constipation and vomiting [64] that can lead to esophagitis or aspiration with aspiration pneumonia. Dysembryogenetic abnormalities including esophageal atresia, imperforate anus, tracheo-esophageal fistula, Hirschsprung disease and predisposition to intestinal malrotation have also been reported, with minor frequency [1].

Almost 30% of patients suffer from congenital anomalies of the kidneys and of the urinary tract (CAKUT) such as bilateral or unilateral renal agenesis, duplicated collecting system and hydronephrosis, cryptorchidism and hypospadias, absent uterus or inguinal hernia [65].

Due to the complexity of the above listed clinical manifestations, daily activities of patients affected by 22q11.2 DS can be seriously compromised [66] and in particular severe forms of CHD contribute to a lower life-expectancy than unaffected population [67,68].

The summary of the principal clinical manifestations described in 22q11.2 DS patients and their percentages of occurrence is listed in Table 1.

## 3. Genetic Features of 22q11.2DS

The 22q11.2 region has a complex structure, characterized by low copy repeats (LCR22A, LCR22B, LCR22C, LCR22D) which share >96% of their sequence and are particularly prone to nonallelic homologous recombination during gametogenesis [21]. In particular, LCR22A region is more susceptible to rearrangements, since it is characterized by hypervariability in the organization and in the copy number of duplicons which is human-specific and potentially variable in the population [73,74]. Depending on LCRs involved, the deletions causing 22q11.2DS may have different sizes and localizations. Almost 85% of patients share the so-called typical deletion of 3 Mb between LCR22A and LCR22D [27]. In patients with the typical deletion, the breakpoints within LCR22A and LCR22D are substantially clustered; they show small differences in genes not directly linked to clinical signs of the syndrome, thus not playing a major role in the variability of 22q11.2 DS [27]. Less frequently, the syndrome is caused by atypical, proximal or distal deletions [1].

In 90% of cases with typical 3 Mb or 1.5 Mb deletions the meiotic error occurs de novo. On the contrary, smaller size proximal or distal deletions are more frequently inherited [75]. These deletions are less penetrant and may be unrecognized since the patients are usually less symptomatic and the deletion cannot be identified using FISH (Fluorescent In Situ Hybridization). CMA (chromosomal microarray) is the most useful genomic testing method that allows to determine the copy number of sequences and to detect the recurrent deletion in a proband. The ability to size the deletion depends on the type of microarray used and the density of probes in the 22q11.2. Cardiovascular manifestation is found in approximately two-thirds of children with 22q11.2DS, so it represents one of the major diagnostic clues for 22q11.2DS [37,42].

FISH with a probe that targets the proximal fragment of the region (LCR 22A–22B) can also be used for the diagnosis. 

**Figure 1:**
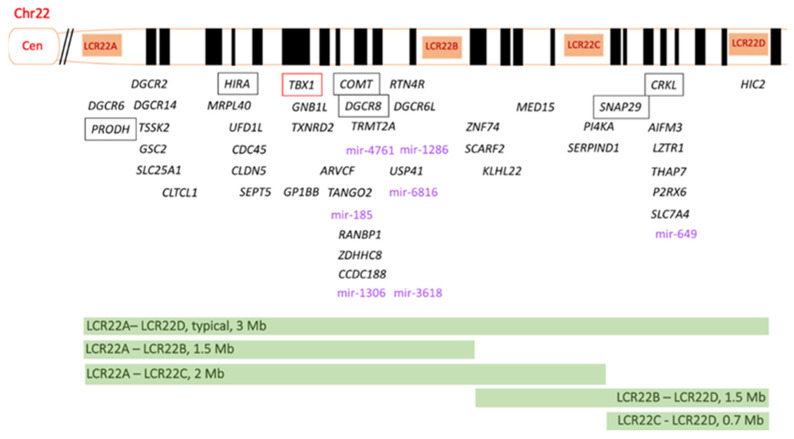
Schematic representation of the 22q11.2 region, including the four low-copy repeats (LCRs) LCR22A-LCR22D. The 46 protein-coding genes are indicated in black. TBX1 (T-box 1) is highlighted in red, since it is considered the main genetic driver of 22q11.2 DS. The potential pathogenetic role of *PRODH*, *HIRA*, *COMT*, *DGCR8*, *SNAP29* and *CRKL* genes (in the box) is discussed in the text. The 7 micro-RNAs are indicated in violet. The size and the localization of the different deletions are shown at the bottom of the figure. Mir, microRNA.

The 3 Mb typically deleted region includes 90 genes: 46 protein-coding genes, 7 microRNAs, 10 non-coding RNAs and 27 pseudogenes [76] (Figure 1 and Table 2).

Among the most studied protein-coding genes, *TBX1* (T-box transcription factor 1), located at the proximal side of the 22q11.2 region, has been shown to play a crucial role in the pathogenesis of 22q11.2DS [77]. TBX1 is implicated in DNA transcriptional regulation, acting on chromatin accessibility through the interaction with histone modifiers and chromatin remodeling complexes, with a direct effect on H3K4me1 levels [78]. TBX1 regulates monomethylation of histone 3 lysine 4 (H3K4me1) through interaction with and recruitment of histone methyltransferases and demethylases. It has been proposed as a priming factor that plays a role in keeping targeted chromatin accessible to other regulatory factors, which may be activators or repressors [78] and it is involved in the regulation of developmental processes [79]. Heterozygous *Tbx1* mouse mutants (*Tbx1*^+/−^) show low penetrance of cardiovascular abnormalities with normal thymus gland, while *Tbx1*^−/−^ knockout is embryonic lethal and mice show abnormal development of pharyngeal arches and pouches [39]. TBX1 is required for the characteristic segmentation of the pharyngeal apparatus in arches and pouches [80]. A strict relationship between TBX1 dosage and retinoic acid signaling pathway during embryonic development has been described [81,82]. The vitamin A active metabolite is a key morphogen involved in pharyngeal apparatus segmentation [83], as demonstrated by teratogenesis evidence associated with its exposure during pregnancy. Likewise, vitamin B12 has been identified as a positive regulator of *TBX1* gene expression. Studies conducted using mouse models have shown that vitamin B12 can partially rescue the haploinsufficiency phenotype [84]. Furthermore, TBX1 finely regulates the interaction between VEGFR2 (vascular endothelial growth factor receptor 2) and VEGFR3 (vascular endothelial growth factor receptor 3) during brain microvascular organization and is implicated in cerebral cortex development [85].

Another protein-coding gene involved in 22q11.2 pathogenesis is *CRKL* (V-crk avian sarcoma virus CT10 oncogene homologue-like). Due to its central role in kidneys and urinary tract development, *CRKL* is considered the genetic driver of CAKUT occurring in 22q11.2DS patients [63]. In patients with “partial DGS”, characterized by a normal or slightly reduced number of T-lymphocytes, CRKL deficiency is involved in the mechanisms leading to impaired T-cell proliferation, something that has been shown even in the absence of lymphopenia [86]. Indeed, proliferative response in 22q11.2DS patients is relatively unaffected when assays are normalized for T-cells, and likewise, standard mitogen proliferation tests are usually impaired due to extremely low T-cell counts [47]. Furthermore, CRKL is required for natural killer cells’ physiological activity, since its haploinsufficiency is associated with the functional deficiency of this lymphocyte subpopulation [87].

Another gene of interest in 22q11.2DS is *DGCR8* (DiGeorge critical region 8). The role of this gene will be discussed in Section 4.1.

*HIRA* (histone cell cycle regulator) regulates gene expression, modulating the incorporation of the H3.3 histone into the chromatinic structure [88].

Evidence suggests that genes deleted in the 22q11.2 region participate in complex networks of interactions influencing, with their altered dosage, a plethora of different signaling pathways. Since 22q11.2 hemizygosity alone does not explain the genetic basis of the phenotypic variability observed in patients, due to the evidence that patients sharing the same deletion present with different clinical phenotypes, additional mechanisms have been proposed. These include epigenetic mechanisms, which are better explained in the following sections. In particular, epigenetic regulation is extremely variable as a consequence of TBX1 hemizygosity, which creates a random epigenetic marking that varies from cell to cell [84,89]. In some, the deletion may unmask recessive mutations in genes located in the intact 22q11.2 region, leading to atypical and more severe presentations of 22q11.2DS [21]. Furthermore, recent evidence demonstrates that 1% of patients with 22q11.2DS may be affected by a second genetic condition in the context of a dual diagnosis [90,91,92].

Recent studies suggest that copy number variants (CNVs) of genes located outside the 22q11.2 region may partially explain the variability and complexity of different phenotypes observed in patients sharing the same deletion, increasing the risk of developing certain pathological manifestations. In 22q11.2DS patients, CNVs of the genes *GPR98* (G-protein-coupled receptor 98) [93], *KANSL1* (KAT8 regulatory NSL complex subunit 1 gene) [94] and *SC2A3* (solute carrier family 2 facilitated glucose transporter member 3) [95] have been described as risk factors for congenital heart anomalies.

Some conditions presenting with a phenotype overlapping 22q11.2DS, but without 22q11.2 region anomalies, have been described. The so-called phenocopies of 22q11.2DS are a useful model with which to investigate the potential role of other regions of the genome in the pathogenesis of the main clinical aspects observed in 22q11.2DS patients. Table 3 summarizes the most common clinical manifestation described in 22q11.2DS patients, compared with those observed in patients with other cytogenetic alterations sharing the DiGeorge-like phenotype. In mice, *HoxA3* (class 1 homeobox gene A3) knockout (*HoxA3*^−/−^*)* reproduces the typical clinical defects of the DiGeorge phenotype [96]. Similarly, mutations in specific *Vegf* isoforms are responsible for the same congenital abnormalities caused by *Tbx1* knockout [97]. Furthermore, Cirillo et al. [98] identified the duplication of 15q11.2 region and the deletion of the 22q13.3 and 14q32.1 chromosomal regions in patients with the DiGeorge phenotype not presenting 22q11.2 deletion. The region on chromosome 15 is involved in Prader–Willi/Angelman syndromes, while deleted genes on chromosomes 22 and 14 participate in immune system functions.

## 4. Epigenetic Mechanisms Implicated in the Syndrome

So far, no single gene has been identified to explain all the features of 22q11.2DS, and therefore the correlation between genotype and phenotype is not fully understood. Among the mechanisms involved in conditioning the phenotypic variability of the syndrome, epigenetics seems to play a role [102]. Epigenetic regulation is expressed through DNA methylation, chromatin variation and noncoding RNAs, particularly micro-RNAs (miRNAs) [99].

### 4.1. Micro-RNA Profile

MiRNAs are small non-coding RNAs that negatively regulate gene expression at the post-transcriptional level, resulting in the degradation of target messenger RNA (mRNA) [103]. The involvement of miRNAs in the pathogenesis of 22q11.2DS is suggested by the fact that the deletion involves, among the other factors, the genes encoding for 7 miRNAs and the *DGCR8* (DiGeorge syndrome critical region gene 8) gene, which plays a crucial role in the biogenesis of miRNAs [99]. *DGCR8* encodes a double-stranded RNA-binding protein that is a component of the microprocessor complex Drosha/RnaseIII, involved in the canonical pathway of miRNAs biogenesis [27]. In fact, DGCR8 deletion leads to the subversion of physiological miRNA expression. This aspect is central for understanding the implications of 22q11.2 deletion on the epigenetic regulation of gene expression on a larger scale in the genome.

**Figure 2:**
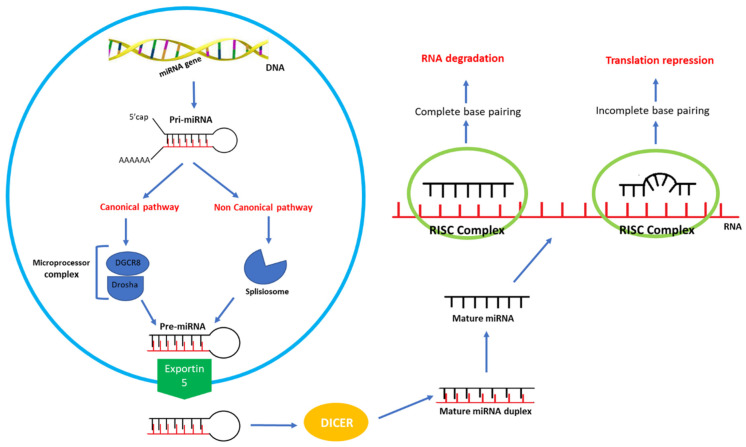
RNA: ribonucleic acid. RISC: RNA-induced silencing complex. DGCR8: DiGeorge syndrome critical region gene 8. The biogenesis of miRNA starts in the nucleus, where RNA polymerase II transcribes miRNA genes into long and capped RNA molecules, called primary miRNAs (pri-miRNAs). The pri-miRNAs can follow two pathways: the canonical and non-canonical pathways. In the canonical pathway, the pri-miRNAs are processed by a complex (microprocessor complex) composed of DGCR8, which works as the noncatalytic subunit, and Drosha, a type of double-stranded RNA-specific endoribonuclease. The microprocessor complex cleaves pri-miRNAs into premature miRNAs (pre-miRNA), which preserve a hairpin structure. In the non-canonical pathway, instead, pri-miRNA are processed by the spliceosome and cleaved into pre-miRNA. Pre-miRNAs are transported into the cytoplasm by Exportin 5. In the cytoplasm, Dicer, an RNase III enzyme, cleaves the hairpin loop and generates a mature miRNA duplex. Subsequently, the mature miRNA duplex separates into two single strands: one is degraded, and the other is incorporated into the RISC complex. In the RISC complex, the mature miRNAs recognize their specific mRNA targets through base pairing, fulfilling the function of transcription regulators. If the base pairing is complete, the mRNA target is degraded; if base pairing is incomplete, mRNA target translation is repressed.

In particular, the microprocessor complex cuts pri-miRNAs, resulting from direct gene transcription, into stem loop structures called pre-miRNAs. The pre-miRNAs are then transferred to the cytosol, where after several steps they are transformed into mature single-stranded miRNAs and guided to their target mRNAs, which are recognized through base pairing [104,105,106,107]. The microprocessor complex plays a crucial role in the canonical biogenesis pathway; the absence of Drosha or DGCR8, in fact, induces the generation of noncanonical miRNAs (Figure 2).

The consequence of the haploinsufficiency of *DGCR8*, therefore, is an alteration in the expression levels of different miRNAs, which can be reduced or increased in affected subjects compared with healthy subjects [108]. Some authors, in addition, correlate miRNA subset expression patterns to specific clinical conditions characterizing 22q11.2 DS. In particular, the upregulation of miR-29 is observed in patients with immunodeficiency; the downregulation of miR-145 is linked to hypocalcemia; the simultaneous upregulation of miR-23 and miR-363 and the downregulation of let-7g are described in association with congenital heart disease [109]. In another study conducted by Stark et al. in 2008, the haploinsufficiency of *DGCR8* is associated with the altered expression profile of some miRNAs, including mir-134, mir-324-5p, mir-491, mir-532 and mir-299, resulting in synaptic alteration and impaired neuronal development [110]. Furthermore, in mouse models, specific miRNA levels have been linked to the size of the hippocampal region of the central nervous system [108,111]. Several genes likely implicated in the pathogenesis of schizophrenia have been identified as targets for miRNA altered by *DGCR8* haploinsufficiency, including *DISC1*, *RELN* and *SYN1*. In another study, *DGCR8* heterozygosity in mice led to a decreased rate of neurogenesis in the adult hippocampus and to altered hippocampus-dependent learning, as well as to the downregulation of some schizophrenia-related genes. Insulin-like growth factor 2 was shown to be able to rescue neurogenesis both in vitro and in vivo, suggesting that impaired adult cell proliferation may contribute to the cognitive deficit schizophrenia found in 22q11.2DS and that it could be “corrected” by IGF2 [112]. As for other experimental therapeutic agents, it has been recently shown that the use of protoporphyrins may play a role in 22q11.2DS patients, increasing miRNA biogenesis in DGCR8 haploinsufficient mouse cells in vitro [113]. It should be noted that often impaired miRNA expression may not be detectable in resting conditions since it may be the result of stress responses [114].

The reduced expression of DGCR8 and a subset of miRNAs has also been described in the forebrain of a heterozygous mouse model in which pyramidal neurons show altered electrical properties [115].

In mouse models, selective Dgcr8 knockout (Dgcr8^+/−,^) leads to behavioral and cognitive abnormalities like hyperactivity and altered spatial working memory [110]. Some authors proposed that 22q11.2 deletion-related miRNA alteration, which could affect genome-wide proteins, may exacerbate altered gene dosage effects of genome-wide rare CNVs (at the level of transcription). miRNA perturbation and CNVs are two well-known pathogenetic elements contributing to the schizophrenia expression risk, and schizophrenia is a typical psychiatric aspect of 22q11.2 DS patients [116]. It has recently emerged that DGCR8 is involved not only in miRNA biogenesis, but also in other processes, such as the repair of UV-induced DNA damage, the promotion of the nucleotide excision of nucleotides, or the regulation splicing process in embryonic stem cells [117,118]. In recent years, several studies have highlighted how miRNAs influence the pathways of physiologic e pathologic stress tissue behavior. In fact, in stress conditions, the upregulation and the downregulation of miRNA expression has direct effects on the mRNA target and cellular response [114].

Among the miRNAs encoded in the deleted 22q11.2 region, miR-185 is certainly the most characterized, especially regarding its targets and their phenotypic implications. SERCA2 is a neuronal regulator of calcium homeostasis, and SERCA2-dependent Ca^2+^ dysregulation has been implicated in several disorders that affect cognitive function, including Darier’s disease, schizophrenia, Alzheimer’s disease and cerebral ischemia. The *SERCA2* gene is a target of miR-185, and its upregulation at the level of excitatory synapses may contribute to the development of cognitive symptoms. miR-185 levels also appear to be crucial for the development of brain abnormalities related to 22q11.2DS [119,120,121]. In the immune system, miR-185 targets are *BTK* in B-cells and *MZB1* in T-cells. A recent study shows that miR-185 overexpression in murine follicular B-cells downregulates *Btk* expression, dampening B-cell receptor signaling. Viceversa, miR-185 downregulation in a Dicer-deficient mouse model is associated with increased *Btk* levels, a skewed BCR repertoire and high titer autoantibodies, suggesting that miR-185 might be implicated in the pathogenesis of autoimmune manifestations [122,123]. Similarly, miR-185 overexpression in thymocytes and peripheral T-lymphocytes in a transgenic mouse model is associated with peripheral T-cell lymphopenia due to impaired T-cell development during the pre-TCR and TCR selection stages. The effect on T-cell development seems to be mediated by the effect of miR-185 on *Mzb1*, *Nfatc3* and *Camk4* expression. Elevations in miR-185 enhanced TCR-dependent intracellular calcium levels, whereas the knockdown of miR-185 diminished these calcium responses. This effect was mediated by *Mbz1*, an endoplasmic reticulum-associated protein that was already known to be implicated in B-cell receptor-driven calcium responses. *MZB1* levels were found to be elevated in thymocyte extracts from several 22q11.2DS patients [124]. Similarly, the overexpression of *Nfact3* and *Camk2d* in the presence of reduced miR-185 levels impairs T-cell development due to their involvement in Ca2+-activated pathways [125,126].

### 4.2. Methylation Profile

Due to the presence in the deleted region of different transcription factor genes, such as *TBX1*, and chromatin remodeling genes, such as *HIRA* [127], some authors have suggested that 22q11.2DS is mainly a transcription deregulation syndrome. The role of TBX1, with a direct effect on H3K4me1 levels, was described in Section 3 [41]. *HIRA* encodes a histone chaperone that preferentially places the variant histone H3.3 in transcriptionally active genes, thus regulating neural progenitor proliferation and neurogenesis [128].

Methyltransferases have been shown to be responsible for episignatures in other neurodevelopmental disorders, such as *DNMT3A* in patients with Tatton–Brown–Rahman syndrome [129] and *KMT2D* in Kabuki syndrome [130]. DNA methylation is an epigenetic modification, regulating chromatin status and gene transcription. Aberrant DNA methylation profiles have often been observed in cancer, and are characterized by the hypermethylation of tumor suppressor gene promoters or methylation defects at imprinted loci [131]. Genome-wide DNA methylation analysis has been used to identify specific episignatures in more than 40 genetic diseases, including some common microdeletion/duplication syndromes [129]. To date, only few studies have been performed to investigate the methylation profile in patients with 22q11.2DS [132,133].

By measuring three histone modifications classically associated with promoter activation status (H3K4me3), enhancer and promoter activation status (H3K27ac), and chromatin opening (H4ac) in CD4 T- and CD19 B-cells, Zhang et al. were able to demonstrate that histone modifications were significantly impaired in both CD4 T-cells and B-cells from 22q11.2DS patients compared with those in controls. The alterations identified were more pronounced in CD4 T-cells. Furthermore, they observed that genes with significantly increased promoter histone modifications in CD4 T-cells from patients with 22q11.2DS significantly overlapped with clusters related to early NFκB and STAT activation and inflammatory responses. By analyzing a cohort of 49 patients with a diagnosis of 22q11.2DS, the authors of [134] were able to identify 160 differentially methylated CpG probes, retained for 22q11.2DS episignature discovery. These probes represent the most differentially methylated CpGs in the cohort when compared with those in the controls; indeed, the selected probes were able to completely separate the 22q11.2DS cases from age- and sex-matched controls.

Moreover, among the patients, they were able to identify two distinct clinical groups based on the methylation profile: patients carrying typical deletions, with a similar phenotype, and those carrying atypical distal deletions, with a milder phenotype. This specific episignature suggests the presence of a common process in the alteration of chromatin remodeling. In addition, methylation alterations in specific imprinted genes and in the MHC (major histocompatibility complex) locus were found to distinguish 22q11.2 individuals with schizophrenia spectrum disorders from those without psychiatric involvement [132]. The role of MHC genes in schizophrenia has been previously reported. Besides their well-studied immune functions, HLA genes contribute to neurogenesis, neural differentiation and migration, synaptogenesis, and synaptic plasticity [135]. An unresolved issue remains in terms of whether or not the DNA methylation signatures found in blood cells reflect the pattern belonging to specific tissues such as the brain. However, it is reasonable thinking that the phenotype of 22q11.2DS could be a result of the combination of the haploinsufficiency of 22q11.2 genes as well as global and specific methylation defects.

## 5. Discussion and Conclusions

Phenotypic features of 22q11.2DS are not fully obvious in patients, and its clinical presentations are remarkably variable. This variability remains largely unexplained. While certain features are more directly related to individual genes, as identified for *TBX1* and *CRKL* regarding cardiac and urinary tract development, other features may require the effects of multigenic reduced gene dosage within the 22q11.2 deletion interacting with permissive variants in modifier genes elsewhere in the genome. Likewise, it is possible that a fine balance deriving from the cooperation between the deleted genes is needed to orchestrate regulatory pathways acting on genome accessibility and transcription. While a role for miRNA in the pathogenesis of the syndrome has been hypothesized, the challenge will be to identify the target genes that are affected by those microRNAs that are found to be dysregulated in 22q11DS and to characterize the pathways of involvement in a much more comprehensive manner. Similarly, further investigation on the effects of the alterations in the DNA and histone methylation profile on gene expression will lead to the identification of potentially targetable networks.

## Figures and Tables

**Table 1:** Schematic representation of the most common clinical manifestations described in 22q11.2DS patients and their percentages of occurrence.

Apparatus Involved	Clinical Features	Percentage	References
Congenital Heart Disease (CHD)	Interrupted aortic arch type B Truncus arteriosus Tetralogy of Fallot Conoventricular septal defects Isolated aortic arch anomaly Double outlet right ventricle Transposition of the great arteries Hypoplastic left ventricle Pulmonary arteries hypoplasia	75%	[1,31,32,33]
Hypocalcemia (hypoparathyroidism)		35%	[69]
Immune Deficiency	Athymia Thymic hypoplasia/ectopy Humoral immunity impairment	50–70%	[70,71]
Craniofacial dysmorphisms	Elongated face Hooded eyelids Epicanthus Wide nasal bridge Short philtrum Micrognathia and retrognathia Low-set small ears	50%	[22,72]
Palatal anomalies	Velopharyngeal insufficiency Overt cleft palate Submucosal cleft palate Bifid uvula	69–100%	[37,38,39]
Renal anomalies	Renal agenesis Multicystic kidney Hydronephrosis Duplicated collecting system	14%	[65]
Skeletal defect	Spine and vertebral anomalies Fingers anomalies	60%	[63]
Learning problems Developmental delay		70%	[42]
Psychiatric disorders	Anxiety Autism spectrum disorders Schizophrenia Behavior disorders Parkinson’s disease	30%	[60,61]
Gastrointestinal abnormalities	Esophageal atresia Esophageal reflux Hirschsprung disease Imperforated anus	30%	[64]

**Table 2:** 46 protein-coding genes located in 22q11.2 region with the associated phenotype, genomic coordinates, and inheritance (omim.org).

	Associated Phenotype	Genomic Coordinates	Inheritance
DGCR6	-	*22:18,906,319-18,912,087*	-
PRODH	Hyperprolinemia type 1	*22:18,912,780-18,936,552*	AR
DGCR2	-	*22:19,036,285-19,122,453*	-
DGCR14	-	*22:19,130,278-19,144,725*	-
TSSK2	-	*22:19,131,307-19,132,621*	-
GSC2	-	*22:19,146,992-19,150,291*	-
SLC25A1	Combined D-2, L-2 hydroxyglutaric aciduria; Presynaptic Congenital Myasthenic Syndrome 23	*22:19,175,580-19,178,735*	AR AR
CLTCL1	-	*22:19,179,472-19,291,718*	-
HIRA	-	*22:19,330,697-19,431,732*	-
MRPL40	-	*22:19,432,544-19,436,074*	-
UFD1L	-	*22:19,449,910-19,479,192*	-
CDC45	Meier-Gorlin Syndrome	*22:19,479,293-19,520,611*	AR
CLDN5	-	*22:19,523,023-19,525,336*	-
SEPT5	-	*22:19,714,502-19,723,318*	-
TBX1	-	*22:19,756,702-19,783,592*	-
GNB1L	-	*22:19,783,222-19,854,873*	-
TXNRD2	Glucocorticoid deficiency?	*22:19,875,521-19,941,817*	-
GP1BB	Bernard-Soulier Syndrome, type B; Giant platelet disorder	*22:19,723,538-19,724,770*	AR AR
COMT	schizophrenia, susceptibility	*22:19,941,771-19,969,97*	AD
ARVCF	-	*22:19,966,726-20,016,822*	-
TANGO2	Metabolic encephalomyopathic crises, recurrent, with rhabdomyolisis, cardiac arrhythmias and neurodegeneration	*22:20,016,999-20,067,163*	AR
DGCR8	-	*22:20,080,240-20,111,871*	-
TRMT2A	-	*22:20,111,871-20,117,253*	-
RANBP1	-	*22:20,116,103-20,127,354*	-
ZDHHC8	-	*22:20,131,803-20,148,006*	-
CCDC188	-	*22:20,148,113-20,151,828*	-
RTN4R	schizophrenia, susceptibility	*22:20,241,414-20,268,317*	AD
DGCR6L	-	*22:20,314,237-20,320,059*	-
USP41	-	*22:20,350,578-20,390,758*	-
ZNF74	-	*22:20,394,150-20,408,454*	-
SCARF2	Van den Ende-Gupta Syndrome	*22:20,424,583-20,437,824*	AR
KLHL22	-	*22:20,441,518-20,497,304*	-
MED15	-	*22:20,507,581-20,587,620*	-
PI4KA	Gastrointestinal defects and immunodeficiency syndrome 2; perisylvian polymicrogyria with cerebellar hypoplasia and arthrogryposis; spastic paraplegia 84	*22:20,707,690-20,858,811*	AR
SERPIND1	Thrombophilia 10 due to heparin cofactor II deficiency	*22:20,774,112-20,787,719*	AD
SNAP29	CEDNIK Syndrome	*22:20,859,006-20,891,213*	AR
CRKL	-	*22:20,917,406-20,953,746*	-
AIFM3	-	*22:20,965,171-20,981,357*	-
LZTR1	Noonan Syndrome 10; Noonan Syndrome 2; Schwannomatosis 2, susceptibility	*22:20,982,296-20,999,031*	AD AR AD
THAP7	-	*22:20,999,103-21,002,117*	-
P2RX6	-	*22:21,009,699-21,028,013*	-
SLC7A4	-	*22:21,028,717-21,032,560*	-
HIC2	-	*22:21,417,370-21,451,462*	-

**Table 3:** Schematic representation of the most common clinical manifestation described in 22q11.2DS patients, compared with those observed in patients with other cytogenetic alterations sharing the DiGeorge-like phenotype.

Clinical Manifestations	10p13-14 DGS2 Locus	3p10.3	4q34.1-35.2	Del2p11.2	Microdup 22q11.2	Del22q13.33 Phelan-McDermid Syndrome
Congenital Heart Disease (CHD)	82%	Yes	15%	No	Yes	3–25%
Hypocalcemia (hypoparathyroidism)	22%	Yes	Na	Yes	Yes	Na
Immune Deficiency	17%	Yes	Na	Yes	Yes	Na
Craniofacial dysmorphisms	50%	Yes	95–99%	Yes	Yes	>75%
Renal anomalies	5%	Yes	Na	No	Yes	38%
Skeletal defects	30–80%	Na	88%	Yes	Yes	>75%
Learning problems/ Developmental delay	80–99%	Yes	65%	Yes	Yes	>75%
Psychiatric disorders	Na	Na	Na	Na	Yes	>75%
Gastrointestinal abnormalities	Na	Na	Na	No	Yes	>25%
**Genes mapping in the region**	Ni			*FOXI3*	See Table 3	*SHANK3*

Na, not available; Ni, *not identified*; DGS2, Di George Syndrome; Del, deletion; Microdup, microduplication; *FOXI3*, Forkhead Box I3; *SHANK3*, SH3 and multiple ankyrine repeat domains 3 [99,100,101].

## Data Availability

Not applicable.
